# Seasonal changes in microbial community composition in river water studied using 454-pyrosequencing

**DOI:** 10.1186/s40064-016-2043-6

**Published:** 2016-04-05

**Authors:** Marija Kaevska, Petra Videnska, Karel Sedlar, Iva Slana

**Affiliations:** Veterinary Research Institute, Hudcova 70, 62100 Brno, Czech Republic; Department of Biomedical Engineering, Brno University of Technology, Technicka 12, 61600 Brno, Czech Republic

**Keywords:** River water, Pyrosequencing, Microbial community, Environmental factors

## Abstract

The aims of this study were to determine the microbial community in five rivers in the proximity of a city in the Czech Republic using 454-pyrosequencing, as well as to assess seasonal variability over the course of 1 year and to identify the factors influencing the structure of bacterial communities. Samples from five rivers around the city of Brno were obtained during four seasons and analysed using 454 pyrosequencing of the 16S rRNA gene. The core composition of bacterial communities consisted of *Actinobacteria*, *Bacteroidetes*, *Proteobacteria*, *Firmicutes*, *Fusobacteria*, *TM7* and others. Our approach enabled us to more closely study the correlation between the abundance of different families and environmental factors. Overall, *Actinobacteria* negatively correlated with phosphorus, sulphate, dissolved particle and chloride levels. In contrast, *Proteobacteria* positively correlated with sulphate, dissolved particle, chloride, dissolved oxygen and nitrite levels. Future work should focus on the dynamics of bacterial communities present in river water and their relation to the overall stability of the water ecosystem.

## Background

The community composition of water microorganisms in natural water bodies is important for the mineralisation of organic matter and circulation of important elements in aquatic environments. Culture-independent methods enable a more complex and detailed view of the structure of bacterial communities in nature. With the development of next-generation sequencing, there have been a large number of studies dedicated to unravelling the structure as well as the function of microorganisms in all kinds of environments. Drinking water and biofilms from water distribution systems have been the subject of such studies (Douterelo et al. 2014; Hwang et al. 2012). There is also a large number of studies focused on waste water (Hu et al. 2012; Ye and Zhang 2013), as well as microbial communities in sea water or estuaries (Gilbert et al. 2012; Zhang et al. 2014). However, only a limited number of studies have focused on the spatio-temporal changes of bacterial communities and their correlation with physical and chemical factors in the environment.

Although there are many studies focused on microbial ecology in different types of water, bacterial communities in urban rivers are not well documented. The majority of studies have focused on indicator bacteria of faecal pollution (Murray et al. 2001). The impact of the chemical and physical properties of water on the structure of bacterial communities has been studied in rivers in China (Liu et al. 2013; Zhang et al. 2012). In Europe, Winter et al. (2007) studied the longitudinal changes in bacterial community composition in the Danube River. The authors found a positive correlation between bacterial richness and concentration of P-PO_4_. However, the bacterial community composition was determined by denaturating gradient gel electrophoresis which did not reveal much information about the species present.

The aims of the present study were to determine the microbial community in five different rivers in the proximity of a city in the Czech Republic using 454-pyrosequencing, as well as to assess their seasonal variability over the course of a year and to identify the factors influencing the structure of bacterial communities.

## Methods

### Sample collection and processing

**Fig. 1:**
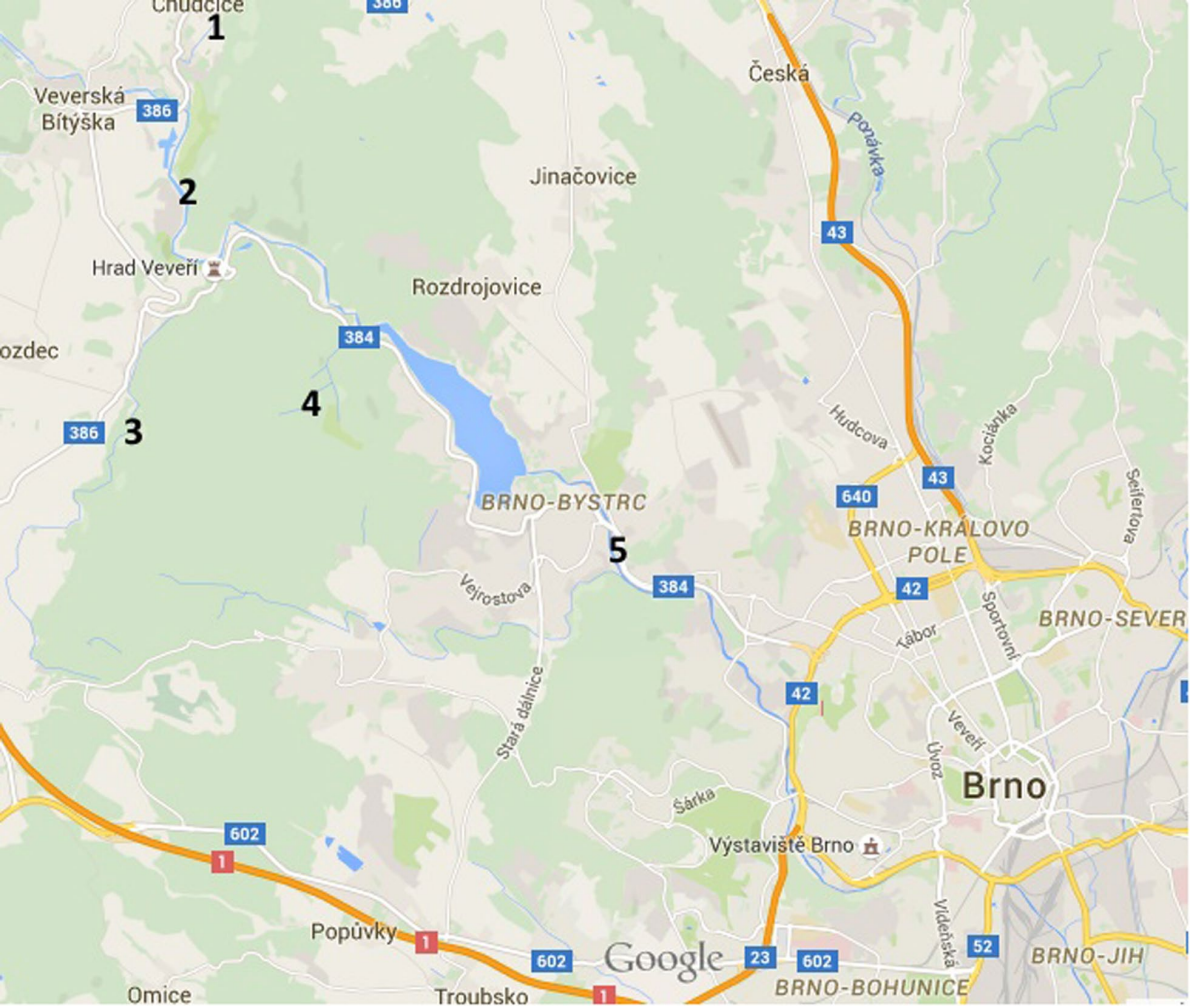
Map with the locations from where the samples were obtained (*1*–*5*)

Samples of 10-L were collected in sterile canisters from the surface water at five sampling points around the city of Brno. The sampling was performed four times during the year 2013, on February 4th, May 3rd, August 5th and November 5th. The exact locations are shown in a map in Fig. 1. The samples were stored at 4 °C for a maximum of 24 h before they were processed. Samples were processed as previously described (Kaevska and Slana 2015). Water was filtered first through pre-filters with pore sizes of 0.8-8 µm and subsequently through 0.22 µm glass fibre filters (Millipore), using a peristaltic pump. The microorganisms from the filter were eluted in 10 ml of PBS buffer with the addition of 0.05 % Tween80 (Sigma) and 3.5 mm glass beads (BioSpec). Samples were vortexed for 5 min, the eluate was subsequently centrifuged at 7000 rpm/5 min, and then the DNA was isolated from the pellet.

### Physical and chemical parameters of water

All of the water samples were subjected to physical and chemical analyses according to accredited methods in a specialised laboratory for water analysis in the company Povodi Moravy a.s. Brno. All the methods performed were according to the ČSN EN ISO/IEC 17025:2005 norms. Analysed parameters included; temperature, pH, oxygen saturation, total oxygen, chlorides, sulphates, nitrates, nitrites, turbidity, total phosphorus, total nitrogen dissolved particles and dissolved oxygen.

### DNA isolation and PCR

DNA was isolated from the pellet using the commercially available PowerSoil DNA isolation kit, MoBio (USA). PCR was performed using universal bacterial primers for amplification of the V2/V3 regions of the 16*S RNA* gene (Nossa et al. 2010). The primers were flanked with standard MID sequences and tags needed for the pyrosequencing. The PCR reaction was prepared using the HotStarTaq Master Mix Kit following the manufacturer´s instructions (Qiagen), with cycling conditions as follows; 95 °C for 15 min followed by 30 cycles of incubation at 94 °C for 40 s, 55 °C for 55 s and 72 °C for 60 s, and a final extension step at 72 °C for 5 min. PCR products were visualised using electrophoresis on 1.5 % agarose gels and purified from the gel using the QIAquick Gel Extraction Kit (Qiagen).

### Pyrosequencing

Amplicon library preparation and high-throughput sequencing was performed using the 454 GS Junior from Roche, using the chemistry recommended by the manufacturer (Roche). Sequences were analysed using the QIIME software (Caporaso et al. 2010). Quality trimming criteria included no mismatch in MID sequences and a maximum of 1 mismatch in primer sequences. The obtained sequences with qual score higher than 20 were shortened to the same length of 350 bp and classified with RDP Seqmatch (Cole et al. 2014) with an operational taxonomic unit (OTU) discrimination level set to 97 %.

Correlations of bacterial occurrence with measured parameters were calculated using Spearman’s rank correlation coefficient. Correlations were visualised using a cluster heatmap generated using MATLAB version 2013a (MathWorks).

## Results

### Environmental characteristics of the water

The environmental variables were measured during the four seasons and are summarised in Table 1. The temperature was consistent among the samples in each season but varied among seasons (range between 1.6 and 20 °C). The pH and oxygen saturation did not greatly vary, but other parameters were highly variable, also within each locality or season (Table 1).

**Table 1:** Environmental characteristics of the samples from five rivers in the four seasons throughout the year

Season	Locality	Temperature (°C)	pH	Oxygen saturation (%)	Chlorides (mg/L)	Dissolved particles (mg/L)	Phosphorus (mg/L)	Nitrogen (mg/L)	Sulphates (mg/L)	Nitrates (mg/L)	Nitrites (mg/L)	Turbidity (ZFn)	Dissolved oxygen (mg/L)
Winter	1	2.7	8.2	99	95.3	683	0.178	4.96	120	0.128	19.5	7.21	12.9
	2	2	7.9	102	54.3	324	0.149	8.81	45	0.145	38.1	17.8	13.6
	3	1.6	7.9	102	93.7	412	0.18	9.81	50.2	0.184	41.6	20.8	13.7
	4	2.2	8.2	104	120	778	0.214	6.45	88.1	0.105	28.6	4.83	13.7
	5	1.7	8.2	97	30.2	249	0.103	7.09	52	0.112	30.2	9.75	13
Spring	1	12	8	93	40	575	0.347	4.65	84.5	0.233	15.2	43.9	9.8
	2	11.3	8	104	28.7	266	0.198	3.52	42	0.115	19.5	44.2	11.1
	3	12	8	99	56	350	0.262	4.95	53.3	0.131	19.2	46.1	10.4
	4	11.5	8.2	98	60.9	557	0.265	4.94	79.5	0.118	18.8	71.8	10.4
	5	12.1	7.7	91	15.9	230	0.12	4.71	36.2	0.171	17.8	10.1	9.6
Summer	1	14.2	8.3	81	33	566	0.472	4	48	0.443	13.5	6.34	8.2
	2	14.5	8.2	97	23.7	248	0.194	3.53	44	0.062	14.1	7.38	9.7
	3	13.6	8.2	100	57.4	400	0.4	4.22	50.8	0.026	17.6	12.2	10.2
	4	14.8	8.4	103	22.6	695	0.599	5.75	55	0.082	24.7	23.5	10.2
	5	20	8.1	71	15.8	197	0.028	5.01	46.1	0.663	10.8	8.08	6.3
Autumn	1	7	8	90	70.5	648	0.278	4.56	98	0.2	18.5	16	10.7
	2	6.8	8.3	96	28	253	0.141	3.87	44.1	0.066	15.1	5.24	11.3
	3	6.2	8.3	100	53.8	362	0.183	5	53.9	0.069	20.5	5.1	11.8
	4	6.9	8	94	92	730	0.553	7.23	91.4	0.174	27.1	8.45	11.1
	5	7.5	8	82	19.6	205	0.053	2.81	44.5	0.099	10	6.52	9.6

### Microbial community composition

We obtained 89,808 sequences of sufficient quality, with a mean number of sequences per sample of 4550. The numbers of OTUs, Observed species, Chao, Shannon and Simpson indexes are shown in Table 2. The diversity was higher during the winter and autumn season in all of the samples analysed.

**Table 2:** Diversity indexes in the examined samples sorted by season

	Location	Observed species	Chao1	Equitability	Shannon	Simpson
Winter	1	1339	2322	0.84	8.71	0.99
	2	2071	3370	0.83	9.19	0.99
	3	1904	3219	0.88	9.58	1.00
	4	1756	2972	0.86	9.25	0.99
	5	1499	2649	0.81	8.55	0.99
Spring	1	842	2072	0.83	8.10	0.99
	2	1108	2893	0.78	7.89	0.98
	3	897	2015	0.80	7.83	0.98
	4	950	2784	0.69	6.83	0.96
	5	672	1779	0.79	7.38	0.98
Summer	1	768	2333	0.66	6.35	0.86
	2	518	2035	0.59	5.31	0.89
	3	803	2005	0.65	6.26	0.94
	4	762	2619	0.64	6.17	0.93
	5	531	987	0.77	7.00	0.97
Autumn	1	1560	3745	0.88	9.31	0.99
	2	1777	5331	0.86	9.25	0.99
	3	1566	3976	0.91	9.63	1.00
	4	1422	3921	0.73	7.61	0.96
	5	918	2248	0.70	6.92	0.95

**Fig. 2:**
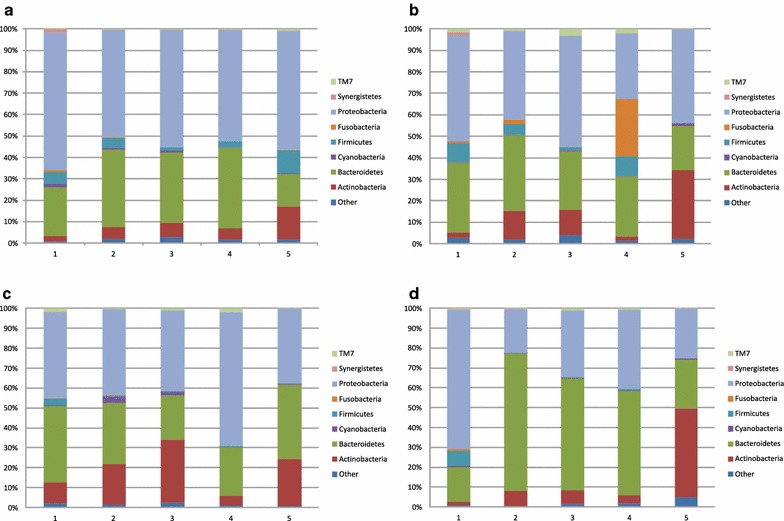
Bacterial community composition in five rivers in **a** winter, **b** spring, **c** summer and **d** autumn

The bacterial community composition in all of the samples on the phylum level is shown in Fig. 2. The core composition of bacterial communities consisted of *Actinobacteria*, *Bacteroidetes*, *Proteobacteria*, *Firmicutes*, *Fusobacteria*, *TM7* and others. In most of the localities the *Proteobacteria* were the most abundant phylum. However, the *Fusobacteria* predominated in one location in spring (Fig. 2). Also, we observed an increased abundance of *Bacteroidetes* in summer and in three localities in the autumn season.

### Statistical correlation

**Fig. 3:**
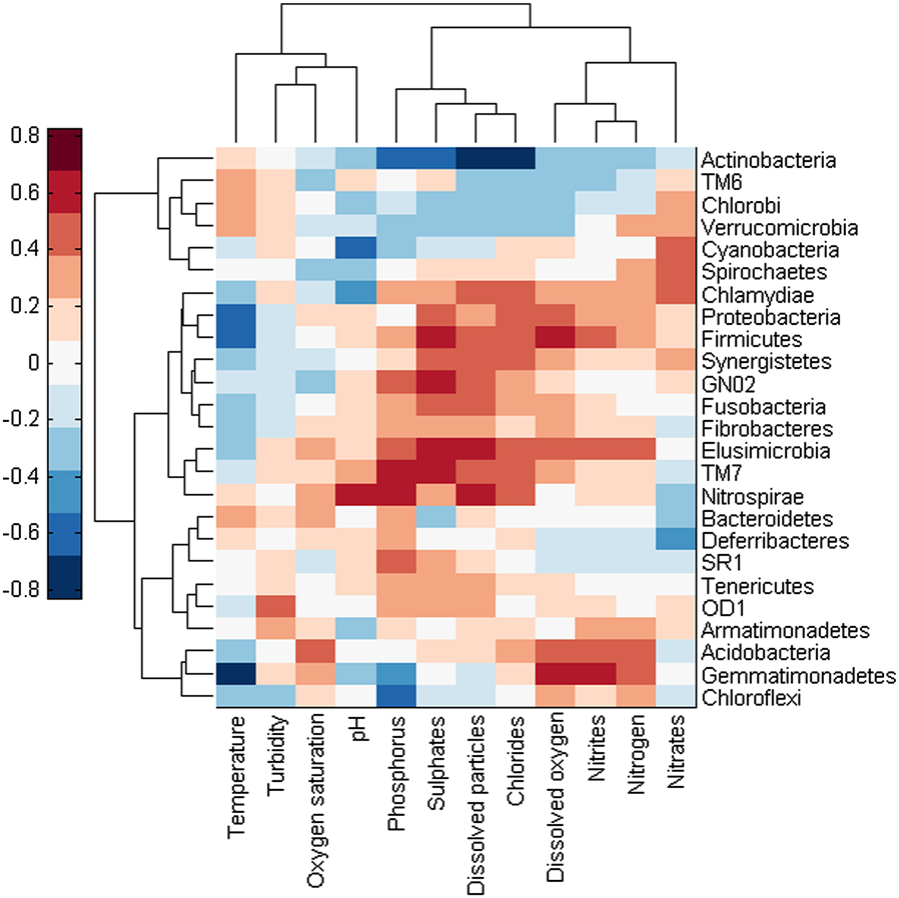
Correlation of environmental factors with abundance of different phyla in river water

**Fig. 4:**
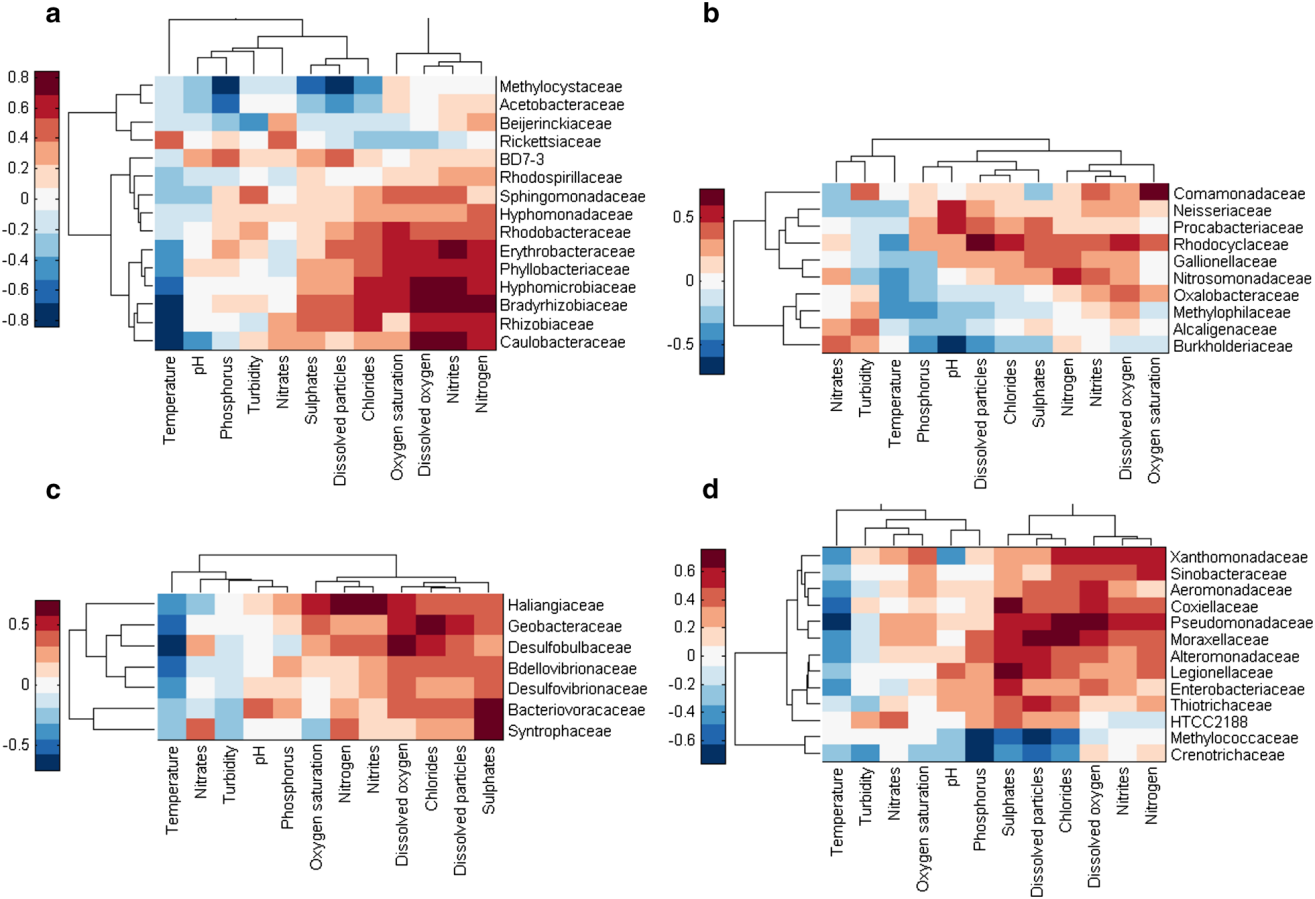
Correlation of environmental factors with abundance of families within: **a** *Alphaproteobacteria*, **b** *Betaproteobacteria*, **c** *Gammaproteobacteria* and **d** *Deltaporteobacteria*

**Fig. 5:**
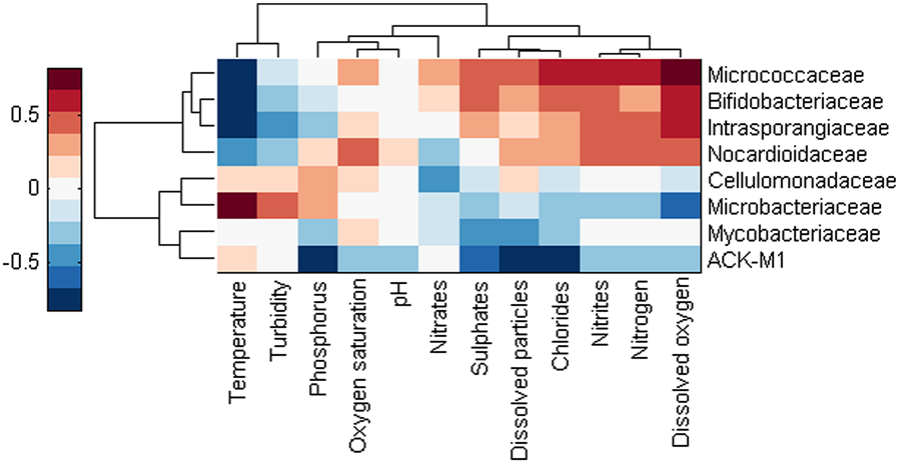
Correlation of environmental factors with abundance of families within *Actinobacteria*

The correlations of the abundance of phyla and families with environmental factors are shown in heat maps in Figs. 3, 4 and 5. On a phylum level (Fig. 3), temperature did not have a strong impact although we observed a negative correlation with the phyla *Gemmatimonadetes*, *Proteobacteria* and *Firmicutes*. *Firmicutes* positively correlated with dissolved oxygen, sulphate, nitrite and dissolved particle levels. We also observed a strong positive correlation between phosphorus and *Nitrospirae* and *TM7*. *Actinobacteria* negatively correlated with phosphorus, sulphate, dissolved particle and chloride levels. In contrast, *Proteobacteria* positively correlated with sulphate, dissolved particle, chloride, dissolved oxygen and nitrite levels. We further analysed the families present in these two phyla and their correlation with environmental factors. In Fig. 4a–d, the families present in the highest proportion of the samples are shown. In the *Actinobacteria* correlation shown in Fig. 5, several families (*Micrococcaceae*, *Bifidobacteriaceae*, *Nocardiaceae* and *Intraspongiaceae*), positively correlated with dissolved particle, chloride, nitrite, nitrogen and dissolved oxygen levels, while these same families negatively correlated with temperature. *Mycobacteriaceae* were not strongly correlated with any of the parameters analysed, although a weak negative correlation between this family and sulphates and dissolved particles was observed.

## Discussion

We have studied the bacterial community composition at five sampling points around the city of Brno during four seasons. Fifteen environmental factors were also measured at each sampling point and the variance of bacterial families was correlated with the fluctuations in these parameters. A similar study was performed by Zhang et al. (2012) in river water in China; however, the authors used denaturating gradient gel electrophoresis (DGGE) to study the bacterial community composition. Although DGGE and pyrosequencing may target different taxonomical units (Vaz-Moreira et al. 2011), pyrosequencing of the 16*S rRNA* gene is a powerful tool with higher capacity than DGGE and enables in-depth study of community composition.

The microbial diversity indices are shown in Table 2. Chao 1 and Shannon values are similar to those obtained by other authors in river water (Zhang et al. 2012). Our results showed the highest diversity in the autumn season, although the diversity during the rest of the year was relatively steady.

Similarly to other studies, we found the bacterial composition to be characteristic for a season rather than a given locality (Gilbert et al. 2012; Zhang et al. 2012, 2014), although we found sampling site 5 to have a different composition (Fig. 2). This may be due to the fact that the sampling site lay downstream of a populated area, at a point where the river is wide.

Our approach enabled us to obtain detailed insight into bacterial community structure and thus we could correlate specific genera with certain environmental factors. More specifically, we performed correlation analyses on the phyla and also on families belonging to *Proteobacteria* and *Actinobacteria*. Similar statistical analyses regarding the influence of environmental factors on microbial communities were performed on samples from a subtropical river (Liu et al. 2013), although the authors used DGGE and could not precisely specify the bacterial genera present. However, the authors found a strong correlation between temperature and variation in bacterial community composition. Another study performed by Zhang et al. (2014) presents further evidence that temperature is the main force driving changes in bacterial composition. We also observed changes in the phyla composition between seasons (Fig. 3), which we studied more closely in *Proteobacteria*, where we observed specific families to be strongly negatively correlated with temperature (Fig. 4). However, another study suggests that other factors, such as salinity or depth could be more important in determining variability (Fortunato et al. 2012). The composition of nutrients is also important for bacterial composition as different organisms are adapted to different conditions. We analysed the correlation between bacterial communities and eleven water factors. All of these had a certain influence on the community composition, although we could not specify which factor exerted the highest influence. Environmental factors were also analysed in relation to bacterial richness along the Danube River; phosphates and chlorides were found to be the most important in this regard (Winter et al. 2007).

## Conclusions

In conclusion, we have analysed the bacterial community composition at five different sites and have determined the correlation with different environmental factors. We have identified a few factors that influenced the composition of bacterial communities in rivers. Fluctuations in the bacterial communities across seasons, and at different temperatures, as well as at different phosphorus and chloride levels were noted. However, we have not focused on the levels of pollution in the water or its effects on the bacterial community composition. Future work should address the dynamics of bacterial communities and their relation to the overall health or pollution levels of the water ecosystem.
